# Can Neutrophil-Lymphocyte Ratio in Complete Blood Count Help in the Differential Diagnosis Between Acute Appendicitis and Right Ureteral Stones in Pediatric Age Groups?

**DOI:** 10.7759/cureus.23866

**Published:** 2022-04-06

**Authors:** Osman Hakan Kocaman, İsmail Yagmur, Tansel Günendi, Mehmet Demir, Adem Tunçekin, Mehmet E Boleken

**Affiliations:** 1 Pediatric Surgery, Harran University, Şanlıurfa, TUR; 2 Urology, Harran University, Şanlıurfa, TUR

**Keywords:** appendicitis, children, ureteral stone, neutrophil lymphocyte ratio, complete blood count

## Abstract

Background and objective

Abdominal pain is one of the most common problems in children presenting to the pediatric emergency departments and is often a diagnostic challenge for the physician. Clinical studies have been carried out on adult patients to differentiate between ureteral stones and acute appendicitis (AA) in which neutrophil-lymphocyte ratio (NLR) and platelet-lymphocyte ratio (PLR) were analyzed in the complete blood count, and it was found that NLR and PLR could help in the differential diagnosis. In this study, we investigated whether complete blood count parameters in pediatric patients could be helpful in the differential diagnosis between AA and right ureteral stones.

Methods

The files of pediatric patients who were followed up and treated for AA and right ureteral stones between January 2019 and March 2021 were reviewed retrospectively. The demographic characteristics of the patients and their WBC, NLR, PLR, and red cell distribution width (RDW) values were evaluated to determine whether there was a difference between the two groups.

Results

In this study, 77 patients with AA and 48 patients with right ureteral stones were included. Univariate regression analysis revealed that age, gender, WBC, PLR, and NLR were factors likely responsible for AA. As per multivariate linear regression analysis, NLR level (odds ratio: 0.407; 95% CI: 0.293-0.566; p<0.001) was an independent predictor of AA.

Conclusion

Based on our findings, NLR can help in establishing the diagnosis in pediatric patients who present to the emergency department with right lower quadrant pain, and in whom physical examination, routine laboratory tests, and imaging methods cannot help distinguish between AA and ureteral stones.

## Introduction

Acute abdominal pain is one of the most common complaints encountered in pediatrics with a wide spectrum ranging from self-limiting conditions like gastroenteritis, and constipation, and those requiring emergency medical treatment like urolithiasis to acute appendicitis (AA) or bowel obstruction that necessitate emergent surgical intervention [1].

AA is diagnosed in 1-8% of children presenting to the pediatric emergency department with acute abdominal pain, and it is the most common condition requiring emergency abdominal surgery in children [2]. However, children with AA often present to the emergency department with nonspecific complaints such as abdominal pain, nausea, or vomiting, which makes it difficult for emergency physicians to confirm the etiology of the symptoms, which may delay the diagnosis [3]. In previous studies, the frequency of perforation was reported to be between 17 and 33% and the rate of negative laparotomy was between 3 and 54% [1]. Since ultrasonography (USG) does not emit radiation, it is one of the most preferred imaging methods in the differential diagnosis of conditions related to acute abdomen, with a specificity rate of 68-90% in children [4]. Intravenous contrast-enhanced CT of the abdomen is often considered for the diagnosis of AA; although there has been a significant increase in the use of CT over the years, the rate of negative appendectomy has not changed significantly [5].

Pediatric urological causes leading to negative appendectomies are rare. On the other hand, urological symptoms now and then accompany the symptoms in patients with AA. More than 30% of these patients have abnormal urinalysis findings [6]. Renal colic is a clinical condition characterized by severe abdominal and flank pain due to urinary obstruction. The main complaint in children with urinary system stones was reported to be abdominal pain, at a rate of 65% [7]. In particular, right ureteral stones can be confused with AA, and the emergency physician may hesitate between AA, which requires surgery, and ureteral stones, which may not require surgery, and this may lead to the patient being inadvertently discharged. Emergency physicians may have difficulties in differentiating between these two clinical entities, especially in rural hospitals where advanced diagnostic tools such as USG and CT are scarce.

The complete blood count is the most common laboratory test performed in patients presenting to the pediatric emergency department with acute abdominal pain. Historically, clinical studies carried out on adult patients that seek to differentiate between ureteral stones and AA in which neutrophil-lymphocyte ratio (NLR) and platelet-lymphocyte ratio (PLR) were analyzed in the complete blood count have found that NLR and PLR could help in the differential diagnosis [8-10]. In our study, we investigated whether complete blood count parameters in the pediatric patient group could be helpful in the differential diagnosis between AA and right ureteral stones.

## Materials and methods

After obtaining approval from the local clinical research ethics committee (Harran University Clinical Research Ethics Committee; Ethics Committee Decision No: 21.05.24), the files of pediatric patients who were followed up and treated for AA and right ureteral stones between January 2019 and March 2021 were reviewed retrospectively. The age, gender, and demographic characteristics of the patients, as well as WBC, NLR, PLR, and red cell distribution width (RDW) values, were examined to determine whether there was any difference between the two groups. Patients who had undergone surgery for perforated appendicitis and patients with other chronic comorbidities were excluded from the study.

Statistical analyses

The SPSS Statistics version 22 statistical program (IBM, Armonk, NY) was used in the analysis of the data. The Kolmogorov-Smirnov test was used to evaluate the distribution. Continuous variables were presented as median (interquartile range 25-75) or mean ± standard deviation, and categorical variables were presented as numbers and percentages. The student's t-test and the Mann-Whitney U test were used to compare continuous variables, and the chi-square test was used to compare categorical variables. Univariate risk analysis was used to identify potential risk factors for AA. Multivariate logistic regression analysis was employed to determine the independent predictor of AA. Receiver operating characteristic (ROC) curve analysis was used to determine the optimal cut-off value for NLR. A p-value <0.05 was considered statistically significant.

## Results

Seventy-seven patients with AA (52 boys, 25 girls) and 48 (28 boys, 20 girls) patients with right ureteral stones were included in this study. There was no significant difference between the groups except for age, which was significantly higher in patients with AA compared to the right ureteral stone group (10.8 ± 3.8 years vs. 7.5 ± 4.2 years; p<0.001).

When laboratory parameters were analyzed, NLR [8.2 (5.2-11.3) vs. 1.7 (0.96-2.8), p<0.001], PLR [201 (160-252) vs. 122 (88-156), p<0.001], and WBC values (15 ± 4 vs. 10 ±3, p<0.001) were found to be significantly higher in patients with AA. When the RDW value was evaluated, no statistically significant difference was found between patients with AA and those with right ureteral stones. Baseline characteristics and laboratory parameters of the study population are presented in Table 1.

**Table 1:** Demographic and laboratory characteristics of the study population NLR: neutrophil-lymphocyte ratio; PLR: platelet-lymphocyte ratio; WBC: white blood cells; RDW: red cell distribution width

Variables	Appendicitis group (n=77)	Right ureteral stones group (n=48)	P-value
Age in years, mean ± SD	10.8 ± 3.8	7.5 ± 4.2	<0.001
Gender (male/female), n (%)	52 (67.5%)/25 (32.5%)	28 (58.3%)/20 (41.7%)	0.297
NLR, median (IQR)	8.2 (5.2-11.3)	1.7 (0.96-2.8)	<0.001
PLR, median (IQR)	201 (160-252)	122 (88-156)	<0.001
WBC (10e^3^/uL), mean ± SD	15 ± 4	10 ± 3	<0.001
RDW (%), mean ± SD	13 ± 1.5	12 ± 1.0	0.012

Univariate regression analysis revealed that age, gender, WBC, PLR, and NLR were factors likely responsible for AA. As per multivariate linear regression analysis, NLR level (odds ratio: 0.407; 95% CI: 0.293-0.566; p<0.001) was an independent predictor of AA (Table 2).

**Table 2:** Univariate and multivariate logistic regression analysis representing the independent predictors of appendicitis PLR: platelet-lymphocyte ratio; WBC: white blood cells; NLR: neutrophil-lymphocyte ratio

Variables	Univariate		Multivariate	
	OR (95% CI)	P-value	OR (95% CI)	P-value
Age	0.815 (0.735-0.903)	<0.001	0.912 (0.788-1.057)	0.221
Gender	0.673 (0.319-1.419)	0.297		
PLR	0.977 (0.969-0986)	<0.001	0.993 (0.976-1.011)	0.471
WBC	0.666 (0.575-0.771)	<0.001	0.883 (0.712-1.095)	0.259
NLR	0.407 (0.293-0.566)	<0.001	0.407 (0.293-0.566)	<0.001

**Figure 1:**
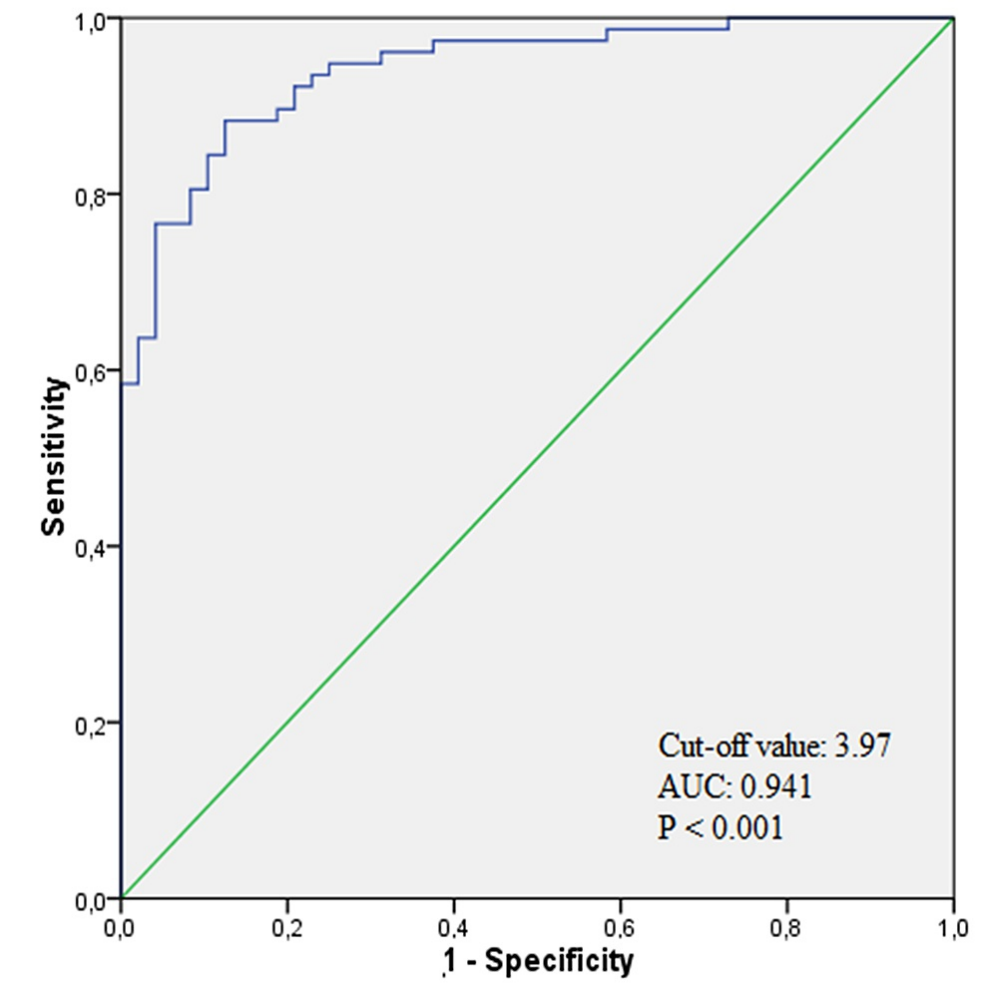
ROC curve of NLR AUC: area under the curve; ROC: receiver operating characteristic; NLR: neutrophil-lymphocyte ratio

When ROC curve analysis was performed to determine the optimal threshold value of NLR to predict AA, NLR ≥3.97 was found to predict AA with a sensitivity of 88% and specificity of 87% (AUC: 0.941; 95% CI: 0.903-0.980; p<0.001) (Figure 1).

While abnormal urinalysis findings were detected in 51.94% (n=40) of the patients with AA, they were found in 83.33% (n=40) of the patients with right distal ureteral stones. When the imaging methods of patients with AA were evaluated, AA was detected in 54 patients only by abdominal USG; in 17 patients, abdominal USG failed to detect AA, and hence the diagnosis was established by abdominal CT. In six patients, AA could not be detected via any imaging methods, and it was diagnosed during explorative laparotomy. Right distal ureteral stones revealed themselves in 31 patients by abdominal CT, by direct urinary system radiography in 12, and by abdominal USG in five patients.

## Discussion

Based on our comprehensive search of the relevant literature in English, we could not locate another study concerning NLR values in children with AA versus those with right ureteral stones. In our study, WBC, PLR, and NLR were determined to be significantly higher in children with AA and we found that NLR was the most useful one for predicting AA among these three parameters.

Abdominal pain is among the most common complaints in children and constitutes approximately 10% of emergency department presentations [11]. AA is the most common cause of emergent abdominal surgery in children. It can be diagnosed by palpating the tenderness that may start around the umbilicus, which then localizes to the McBurney point, as well as based on leukocytosis and high C-reactive protein (CRP) value in laboratory studies and positive abdominal USG findings.

Since the diagnosis of AA is difficult, especially in young children, it has been reported that perforation rates are higher in them compared to adults (30-65%) [12]. One of the diseases that can be confused with AA is right ureteral stones. The incidence of urolithiasis in children is around 6-10%, and a majority of them are benign ureterolithiasis [13]. Paajanen et al. [14] reported a 16% incidence of sensitivity in McBurney point in patients with right-sided renal colic and urological causes leading to negative appendectomy, which was found to be 1-4%. They also concluded that pain in ureteral stones usually reveals itself as colic type in the flank or lower abdomen region. This can be explained by the local inflammatory reaction induced by an embedded stone in the pelvic part of the ureter. Therefore, additional examinations and imaging methods are needed to differentiate between AA and right ureteral stones.

AA occurs due to the obstruction and consequent inflammation of the appendix vermiformis. In this inflammatory response, neutrophils first accumulate in the bloodstream [15]. While inflammation is not yet seen in the acute phase of renal colic, inflammatory processes develop through mediators such as prostaglandins and nitric oxide [16]. WBC is the most commonly used laboratory test in the diagnosis of AA. In previous studies, the sensitivity of WBC in the diagnosis of AA was reported to be 71.2-87.1% and the specificity was found to be 67.2-91.7% [17]. In our study, we also found WBC to be significantly higher in patients with AA. Nevertheless, WBC was not an independent determinant in the differentiation of AA and right ureteral stones.

Some studies have shown that high RDW levels can be seen in various pathological conditions. However, in studies on the differential diagnosis of AA and determining the severity of appendicitis in children, a superiority of RDW over other parameters has not been demonstrated [17,18]. In studies conducted on adults, RDW was not found to be significant in the differential diagnosis between AA and ureteral Stones [8,9]. In our study, RDW was not statistically significant in the differentiation between AA and right ureteral stones.

The secretion of cytokines is increased at the beginning of inflammation in platelets like neutrophils, and increased cytokines contribute to the increase of inflammation by increasing the synthesis of new neutrophils and platelets. PLR has been used to evaluate the inflammatory process leading to the release of pro-inflammatory cytokines and the proliferation of megakaryocytes [19]. In a study published by Bekdas et al. [20], the sensitivity was 61.8% and specificity was 62.5% for PLR in the differentiation of simple versus complex appendicitis in children. Sönmez et al. [8], in their study differentiating AA from renal colic in adult patients, found the sensitivity of PLR for AA to be 60.4% and specificity to be 56%. In our study, the PLR value was significantly higher in the AA group; however, in the multivariate linear regression analysis, it was not as reliable as NLR in differentiating AA from right ureteral stones.

NLR is a useful and simple marker of inflammation that can be easily calculated on a complete blood count [21]. In inflammatory stress conditions such as AA, leukocytes in the bone marrow shift to neutrophil production, while the number of lymphocytes decreases relatively. The ratio of these two values can be interpreted as showing the adequacy of the cellular immune response against this situation, despite the severity of the systemic inflammation. Yazici et al. [15] showed that NLR greater than 3.5 had maximum sensitivity and greater sensitivity than the number of WBCs in a group of pediatric appendicitis patients. Prasetya et al. [22] found that NLR showed high accuracy in the diagnosis of AA and in differentiating complicated appendicitis from simple appendicitis. There are a few studies showing the use of NLR in the differentiation of AA from renal colic, and these studies were performed in the adult patient population [8-10]. In a study among adult patients, Sönmez et al. [8] found a sensitivity of 72%, a specificity of 63.5%, and a cut-off value of 3.67% for NLR in the differentiation between AA and renal colic. A study by Chen et al. [10] reported the specificity of NLR to be 81.82% and sensitivity to be 68.87% in the differentiation between AA and right ureteral stones in patients. In our study, the cut-off value for NLR was found to be 3.97, and we found that this value predicted AA with a sensitivity of 88% and a specificity of 87%.

In the study by Moosmann et al. [23], they determined the normal values for NLR and PLR in the pediatric population. In our study, NLR and PLR were found to be much higher than normal values in patients with AA, while they were found to be close to normal values in patients with right ureteral stones. In the light of these data, we determined that NLR ≥3.97, which can be easily be derived from complete blood count, has an independent predictive value in the differentiation between AA and right ureteral stones in pediatric patients.

Limitations

This study has a few limitations. Although we adjusted for various risk factors in the multiple analysis, we could not exclude the possibility of residual confounding effects from unmeasured covariates such as CRP. We could not include it in this study because the CRP value was not measured in most patients in the ureteral stone group.

## Conclusions

We believe our study is the first of its kind on the use of NLR in children for the differentiation between AA and right ureteral stones. We think that NLR will help establish the correct diagnosis in pediatric patients who present to the emergency department with right lower quadrant pain, and in whom physical examination, routine laboratory tests, and imaging methods cannot help to distinguish between AA and ureteral stones.
